# Mobile phone dependence, internet credit use, and subjective well-being

**DOI:** 10.3389/fpsyg.2026.1951029

**Published:** 2026-09-30

**Authors:** Linghui Yang, Jing Jian Xiao

**Affiliations:** 1 Institute for High-Quality Development of Old Revolutionary Base Areas in the New Era, Jiangxi University of Science and Technology, Ganzhou, China; 2 Department of Human Development and Family Science, University of Rhode Island, Kingston, RI, United States

**Keywords:** educational attainment, internet credit use, mobile phone dependence, moderated mediation effect, subjective well-being

## Abstract

The widespread adoption of smartphones and the expansion of digital consumer finance have drawn research attention to their links with individual well-being. While separate strands of literature have examined mobile device usage and digital financial behaviors in relation to life satisfaction, few studies have examined whether excessive mobile dependence is indirectly associated with subjective well-being via financial channels. Using data from the 2021 China Household Finance Survey (CHFS), this study constructs a moderated mediation framework to investigate this indirect pathway and its moderation by educational attainment. Results show that mobile phone dependence is negatively associated with subjective well-being, and that internet credit use statistically accounts for a portion of this association. The mediation is more pronounced among individuals with higher educational levels, and the negative association is concentrated among those with low-risk preference and low-risk asset holdings. These findings offer empirical evidence for research on digital behaviors and well-being and highlight the need for differentiated regulatory approaches that account for variations in educational and financial risk profiles.

## 1 Introduction

Driven by the rapid iteration of information technology, the global smartphone penetration rate keeps rising steadily. Mobile internet has become a primary carrier for individuals to acquire daily information, conduct social interaction, complete online consumption, and access diversified financial services. Mobile phone dependence has been prevalent among consumers across all age groups, substantially correlating with individual daily decision-making patterns and long-term psychological states.

Existing academic discussions have not reached consistent conclusions on the correlation between smartphone usage behavior and subjective well-being. Relevant empirical evidence presents contradictory results across different research samples and research dimensions. From the perspective of problematic smartphone usage, smartphone addiction among college students shows a significant negative correlation with subjective well-being (Su and He, 2024). Likewise, the expansion of digital infrastructure correlates with a continuous decline in self-rated happiness among rural consumers (Fang et al., 2026). Nevertheless, plenty of studies report opposite findings focusing on rational digital usage scenarios. Longer duration and higher frequency of smartphone use correspond to better subjective well-being among adolescent groups (Marciano et al., 2022). Mobile internet use is positively linked to the subjective well-being of elderly consumers in China (Lu and Kandilov, 2021). General internet use contributes to improved consumer well-being (Shuai et al., 2025), and digital embedding can also lift the subjective well-being levels of rural farmers (Chen et al., 2026).

Existing empirical evidence presents contradictory findings on the relationship between digital device usage and subjective well-being. One strand of literature documents negative associations, particularly when smartphone use becomes problematic or excessive (Su and He, 2024; Fang et al., 2026). Another strand reports positive associations, especially when usage is functional, goal-oriented, or serves utilitarian purposes (Marciano et al., 2022; Lu and Kandilov, 2021). These apparently conflicting results can be reconciled by distinguishing between qualitatively different modes of use. Active, purposeful use—such as information seeking, social communication, and instrumental tasks—tends to enhance social capital and life satisfaction. In contrast, passive consumption—such as aimless browsing, short-video scrolling, and prolonged entertainment viewing—may correlate with upward social comparison, diminished self-regulation, and negative affective states. Similarly, utilitarian use (e.g., mobile payment, online banking, information searching) may facilitate daily life and improve efficiency, whereas hedonic use (e.g., gaming, entertainment, recreational browsing) may correlate with time displacement and diminished real-world engagement. This distinction helps explain why studies that aggregate all forms of usage without differentiating their nature often yield inconsistent or even contradictory conclusions.

Prior studies have separately explored the correlation of mobile phone use and digital financial behaviors with subjective well-being, whereas two clear research gaps still exist in the current literature. First, most existing studies concentrate merely on the direct association between mobile phone dependence and subjective well-being, with limited discussion on the potential internal transmission path originating from digital financial behaviors. Few empirical works further verify the mediating linkage of internet credit use between mobile phone dependence and subjective well-being, failing to unpack the hidden financial behavioral pathway behind such a correlation. Second, existing heterogeneous research mostly focuses on conventional group characteristics such as age and urban–rural division, while neglecting the moderating role of educational attainment in the above mediating pathway. In addition, relevant research rarely discusses the boundary conditions of the core correlation from the perspective of individual risk preference and household risk asset holdings. It remains unclear how personal financial risk attitudes and household asset structures adjust the interactive relationship among mobile phone dependence, internet credit use, and subjective well-being.

Given the above backgrounds, grounded in Behavioral Decision Theory and Uses and Gratifications Theory, this study adopts cross-sectional data from the 2021 China Household Finance Survey (CHFS) and establishes a moderated mediation model. It aims to examine the direct correlation of mobile phone dependence with subjective well-being, the mediating role of internet credit use, and the moderating role of educational attainment. This study also conducts heterogeneity tests from the perspectives of individual risk preference and household risk asset holding ratio, so as to fill the above research gaps.

This study makes several marginal contributions to current literature. First, a full financial behavior transmission chain connecting mobile phone dependence and subjective well-being is sorted out, with internet credit use emerging as a statistically consistent intermediate factor in the empirical pattern. Most prior work merely discusses direct correlations between smartphone engagement and personal life satisfaction yet ignores financial behavior routes that carry indirect links between the two variables. With data from the 2021 China Household Finance Survey (CHFS), coherent empirical evidence is offered to explain the hidden drop in personal welfare brought by over-reliance on mobile devices through online borrowing, filling the shortage of targeted discussion on financial transmission routes in relevant fields. Second, the analytical framework of boundary restrictions linking digital usage and subjective well-being is expanded by incorporating educational attainment as one conditional dimension. Risk preference and risky asset holdings are further brought into analysis to form multi-layer heterogeneous research logics. Rarely do past studies distinguish divergent welfare fluctuation patterns among groups with distinct risk features. The establishment of such multi-level heterogeneous structures deepens theoretical cognition regarding differentiated welfare distribution across various population subgroups.

The rest of this study is arranged as follows. Section 2 establishes the theoretical framework and puts forward research hypotheses. Section 3 elaborates the research methodology, including data selection, variable definitions, and empirical model specifications. Section 4 reports all core empirical results. Section 5 further discusses the empirical outcomes, heterogeneity evidence, and robustness findings. Section 6 summarizes core conclusions, provides policy implications, acknowledges research limitations, and proposes future research agendas.

## 2 Theoretical framework and hypotheses

### 2.1 Direct association between mobile phone dependence and subjective well-being

Empirical studies on digital device usage and subjective well-being have produced mixed results. To make sense of these inconsistencies and guide our hypothesis development, this study draws on two complementary theoretical lenses: Behavioral Decision Theory and Uses and Gratifications Theory. Behavioral Decision Theory rests on the premise of bounded rationality (Takemura, 2021). Prolonged exposure to smartphone ecosystems distorts personal cognitive evaluation and diminishes self-restraint capacity, leading to biased financial choices that alter subjective perceptions of life quality. Uses and Gratifications Theory adopts a user-centric demand perspective (Ruggiero, 2000). Balanced, intentional digital engagement fulfills diverse psychological needs and brings positive mental returns; continuous over-dependence on mobile equipment, however, cannot sustain long-term intrinsic demand satisfaction and creates lasting negative psychological responses. Combined, these two theoretical viewpoints establish the foundational logic for hypothesis development in this analysis.

Previous work has often reported inverse links between digital participation and personal subjective well-being. More recent studies have tried to resolve these conflicting findings by distinguishing between usage patterns and access technologies. The association between online activity and life satisfaction differs sharply based on whether digital engagement constitutes problematic overuse. Negative connections only emerge among groups with unregulated digital routines, while measured, reasonable online participation correlates with improved subjective well-being (Donoso et al., 2021). Different network access channels also produce differentiated results: fixed broadband access carries stronger positive connections to life satisfaction relative to mobile cellular networks (Tan et al., 2024). These fragmented results show simple linear models cannot resolve the complicated relationship between heavy mobile device reliance and subjective well-being, and the underlying internal transmission pathway requires rigorous empirical testing.

In addition, deep connections exist between heavy mobile device reliance and digital financial participation, which complicates the association between mobile device usage and subjective well-being. Mobile payment, an embedded cashless settlement function on smart devices, removes the restraint of physical currency transactions and easily stimulates impulsive, excessive consumption that lowers personal life satisfaction (Wang et al., 2022). Digital financial services nevertheless display two-sided characteristics when shaping individual financial perceptions. The adoption of digital finance weakens the tight bond between household financial behaviors and self-evaluated financial status and mitigates the inverse association between accumulated financial pressure and perceived financial stability (Mahdzan et al., 2023).

The two-sided nature of mobile internet activity shows heterogeneous links to personal subjective welfare, a pattern consistent with the core arguments of Uses and Gratifications Theory (Ruggiero, 2000). Regulated mobile internet access lifts life satisfaction among aging cohorts across China (Lu and Kandilov, 2021). Negative interconnections only take shape when mobile device use evolves into unconstrained over-reliance, whereas moderate daily mobile usage aligns with higher subjective well-being (Donoso et al., 2021). Integrating core deductions from Behavioral Decision Theory, Uses and Gratifications Theory, and existing empirical evidence, unregulated, long-term heavy mobile reliance relates to sustained adverse psychological changes that relate negatively to personal life satisfaction and overall subjective well-being. In view of the above theoretical reasoning and existing empirical evidence, this study proposes the first hypothesis:

*H1*: Mobile phone dependence is negatively correlated with subjective well-being.

### 2.2 Internet credit use as a mediator

Behavioral Decision Theory and Uses and Gratifications Theory offer different but complementary insights into the relationships we propose. The former explains the cognitive and behavioral consequences of dependence, while the latter clarifies the motivational processes that give rise to it. According to Uses and Gratifications Theory, individuals actively choose to engage with mobile devices because these behaviors serve specific needs—such as information seeking, social interaction, entertainment, and convenience (Takemura, 2021). When these needs are repeatedly satisfied through instant mobile access, habitual usage patterns form and intensify, gradually evolving into mobile phone dependence. Behavioral Decision Theory then explains the downstream decision-making consequences of such dependence. According to Behavioral Decision Theory, human decision-making is bounded by cognitive limitations and susceptible to perceptual biases (Takemura, 2021). Persistent mobile phone dependence is associated with impaired self-regulation, reduced cognitive control, and heightened sensitivity to immediate rewards (e.g., convenience, instant gratification) while discounting long-term consequences (e.g., debt accumulation, financial stress). This cognitive state makes dependent users more vulnerable to impulsive financial decisions—such as clicking on credit advertisements and applying for online loans—when exposed to digital credit marketing. Thus, Uses and Gratifications Theory explains why individuals become dependent on mobile devices (the motivational pathway), while Behavioral Decision Theory explains how this dependence shows empirical correlations with changes in financial decision-making (the behavioral pathway). Together, these two perspectives jointly suggest an indirect empirical pattern linking mobile phone dependence to subjective well-being through internet credit use. This integrated logic also informs the moderating hypothesis: because educational attainment is associated with higher financial literacy and stronger digital information processing capabilities, the indirect pathway is expected to be more pronounced among highly educated individuals, who are more adept at navigating digital financial platforms and more responsive to online credit information.

Behavioral Decision Theory starts from the observation that human judgment falls short of full rationality. Cognitive biases and varying risk attitudes consistently shape how individuals make everyday choices (Takemura, 2021). Following this theoretical thread, sustained heavy mobile phone use is associated with changes in cognitive perception and self-control capacity, forming an implicit behavioral chain that connects digital daily habits to subjective well-being.

Digital finance plays a vital role in breaking the barriers of traditional financial services and advancing financial inclusion (Showkat et al., 2024). A wealth of prior studies verify that digital activities connect with personal welfare via varied intermediate transmission paths. Nie et al. (2021) observe that smartphone use shifts household income structures, and farming income alongside non-farming income jointly act as transitional connectors between mobile device behavior and life satisfaction. For ordinary internet users, online participation improves subjective welfare by boosting self-confidence, expanding access to health resources, and generating extra non-agricultural employment opportunities (Shuai et al., 2025). From the perspective of cross-contagion in financial risks, internet coverage raises household chances of credit default. Calculated at sample average levels, households with internet access bear default risks 0.474 times larger than households without online access (Donou-Adonsou and Basnet, 2019). Other empirical records identify steady inverse relationships between all types of household debt and subjective well-being (Xiao et al., 2021). An et al. (2025) further elaborate parallel multi-channel transmission logic, proving the positive relationship between mobile payment usage and financial anxiety takes shape simultaneously through overspending and excessive household debt.

On the supply side of online credit products, big data infrastructure supports the steady operation of internet lending platforms in four distinct ways. Massive data resources expand the reach to potential borrowers, accurate risk prediction cuts running costs for lending institutions, customised service packages match diversified market demands on digital financial platforms, and complete data protection rules secure long-term stable credit operations (Zhang et al., 2015). Mature digital lending frameworks are associated with substantially lower barriers to online borrowing. People with heavy mobile phone reliance spend extended time on mobile internet channels, where loan advertisements and instant loan entry windows constantly appear. Unrestricted access to online credit information weakens individual self-regulation and drives impulsive online borrowing behaviors.

Low-barrier digital credit channels also come with hidden financial threats. Rapid digital industrial development enables consumers to obtain expensive short-term credit more conveniently, raising the possibility of rolling household debt growth (Chen, 2020). Consistent with such risk reasoning, the rapid expansion of internet credit has created pervasive debt burdens for households. Repeated excessive online borrowing brings lasting negative psychological and social impacts, with younger groups facing more severe adverse outcomes (Ma and Yao, 2023). Regular online credit engagement accumulates recurring repayment pressure and long-run financial worries, which weaken the sense of life satisfaction.

Synthesising the above theoretical reasoning and accumulated empirical evidence, internet credit participation forms a core transitional bridge linking mobile phone reliance and subjective well-being. Hypothesis 2 is derived from the full set of logical deductions above. Accordingly, this study puts forward the second hypothesis:

*H2*: Internet credit use statistically accounts for part of the negative association between mobile phone dependence and subjective well-being.

### 2.3 The moderating role of educational attainment

Uses and Gratifications Theory establishes the motivational starting point, namely the formation process of mobile-phone dependence, while Behavioral Decision Theory explains the subsequent pathway leading to financial behaviors and subjective-well-being outcomes. This forms the basis for Hypothesis 2 concerning the mediating effect. Further drawing on the bounded-rationality logic of Behavioral Decision Theory, education-related cognitive heterogeneity is associated with conditional differences within this indirect pathway, which supports another assumption that will be constructed in this study. Education may relate to this pathway in two opposing directions. On the one hand, more years of schooling are associated with better financial literacy and risk awareness, which could correspond to less impulsive borrowing. On the other hand, higher education can breed overconfidence in one’s debt management skills and, by lowering barriers to credit access, make online borrowing more readily available. For individuals already dependent on mobile phones, this combination may be associated with underestimating the long-term costs of internet credit. The net effect, therefore, is that the mediating pathway is expected to be stronger among the more educated.

Behavioral Decision Theory’s bounded rationality logic supplies fundamental theoretical support for taking educational attainment as the conditioning variable. Varied schooling backgrounds form systematic gaps in cognitive screening, self-regulation, and risk judgment capacity, which will shift the tightness of the transitional route running from mobile device reliance to subjective well-being through internet credit engagement. Such cognitive heterogeneity across educational strata can fully explain the stratified differences of this indirect transmission chain.

Variations in educational credentials adjust the intensity of the above transitional pathway from the lens of individual cognitive divergence, and such layered patterns can be reasonably interpreted via the analytical logic of Behavioral Decision Theory. A large body of empirical studies has verified stable correlations between educational attainment and individual subjective welfare. Higher educational attainment is closely associated with improved life satisfaction (Jin et al., 2020), while progressive improvement in academic qualifications corresponds to better subjective welfare status (Dong et al., 2025). Individuals with higher educational experience generally maintain higher levels of subjective well-being than those without college education, with educational background serving as a valid predictive indicator of personal welfare status (Qin et al., 2025). Academic cultivation optimizes individual cognitive structures and comprehensive competencies, which also facilitates the iterative development of digital financial services (Lee et al., 2025).

Existing evidence further suggests that digital financial involvement is associated with lower subjective well-being, with increased household debt burdens and continuous overconsumption identified as contributing factors (Meng and Xiao, 2023). The correlation between digital financial participation and subjective well-being exhibits obvious group disparities in terms of educational stratification, urban–rural division, and consumption patterns (Wang and Li, 2026). Individuals with proficient digital information processing skills can operate mobile payment and mobile banking services without technical barriers (Ullah et al., 2022). Recent multi-channel transmission research supplements relevant theoretical logic: the positive correlation between mobile payment usage and financial anxiety is jointly driven by overconsumption and excessive indebtedness, which constitutes a parallel indirect pathway. Financial literacy adjusts the correlation between financial behaviors (overconsumption and excessive online borrowing) and financial anxiety, whereas financial education shapes the two sets of linkages between mobile payment usage and overconsumption, as well as between overconsumption and subsequent financial anxiety (An et al., 2025).

Relevant research confirms that educational stratification brings systematic differences in subjective welfare status and behavioral responses to digital financial products. Life satisfaction varies significantly across groups with different academic backgrounds; individuals with undergraduate education tend to report better subjective well-being compared with those holding junior college and postgraduate qualifications (Su and He, 2024). Furthermore, the statistical correlation derived from digital embedding presents distinct stratified characteristics, with more prominent group differences among western rural populations, low-income groups, basic-education groups, and young and middle-aged populations (Chen et al., 2026).

Educational accumulation enhances digital literacy and the capacity to process complex financial information, resulting in heterogeneous behavioral responses to mobile financial marketing content. Compared with groups receiving limited education, highly educated groups demonstrate stronger capabilities in retrieving, screening, and judging online financial information through mobile devices. Advanced digital operational competencies support smoother usage of mobile payment and online banking functions (Ullah et al., 2022). Mastery of professional financial knowledge acts as a core determinant of fintech product accessibility, while the depth of understanding of financial rules largely shapes individual participation in digital financial businesses (Hasan et al., 2023).

Groups with higher educational attainment are more sensitive to online credit marketing information disseminated via mobile terminals, with a higher likelihood of subsequent impulsive online borrowing. Accordingly, advanced educational background is associated with a stronger indirect pathway from mobile phone dependence to subjective well-being via internet credit use. By contrast, groups with lower educational attainment face prominent operational and cognitive barriers in accessing and using digital credit services, which corresponds to a weaker above indirect transmission linkage. Accordingly, this study puts forward the third hypothesis:

*H3*: Educational attainment moderates the indirect empirical pattern, in which the indirect association between mobile phone dependence and subjective well-being via internet credit use is stronger among consumers with higher educational attainment.

## 3 Methodology

### 3.1 Data and sample

The empirical data used in this research come from the 2021 China Household Finance Survey (CHFS), a nationwide authoritative micro-level survey covering demographic characteristics, daily mobile phone usage behaviors, digital financial activities, and individual psychological status across China. The survey adopts stratified sampling and multi-stage sampling methods to ensure the representativeness and randomness of the overall sample. The original survey covers a total of 22,027 household samples. For sample screening, observations with missing values on core variables, extreme abnormal values, and invalid questionnaire responses are excluded to guarantee data validity. After strict data cleaning, a final valid sample of 14,453 observations is retained for follow-up empirical tests. Importantly, the 2021 CHFS wave is the most recent publicly available release and the first to include questions on both internet credit use frequency and daily mobile phone activity duration. This provides a rare empirical opportunity to investigate how mobile behavior relates to financial decision-making and, ultimately, subjective well-being.

### 3.2 Variables measurement

#### 3.2.1 Dependent variable

The dependent variable is subjective well-being. This indicator is measured directly according to one original single question from the 2021 CHFS questionnaire. The core survey question asks respondents about their overall life satisfaction. A five-point ordered rating scale is adopted for subjective evaluation, where the original values range from 1 for very satisfied, 2 for satisfied, 3 for neutral, 4 for dissatisfied, to 5 for very dissatisfied. In the raw data, higher numeric values correspond to lower levels of subjective well-being. Before formal regression estimation, this original scale is reversed to unify the interpretative logic of all model outputs. After the reversal operation, larger numerical values correspond to higher levels of subjective well-being.

#### 3.2.2 Independent variable

The independent variable adopted in this study is mobile phone dependence. This composite index is compiled based on 11 original questionnaire items concerning consumers’ mobile phone usage behaviors and usage duration from the 2021 CHFS dataset. These items cover mainstream daily mobile phone usage scenarios, which can fully reflect consumers’ overall mobile phone reliance level. The 11 measurement dimensions cover daily social chatting, news browsing, online video viewing, mobile gaming, online reading, mobile shopping, catering and travel reservation, mobile payment, online financial information searching, internet credit page browsing, as well as short video entertainment. All items adopt an 8-point ordered scale, with 0 representing no relevant mobile phone use and 8 standing for the longest usage duration. The final phone use time index is calculated by summing scores of the 11 items, with a theoretical maximum value of 88. Higher total scores correspond to longer daily mobile phone usage time and stronger mobile phone dependence among respondents.

#### 3.2.3 Mediating and moderating variables

The mediating variable is internet credit use. This variable is measured by a retrospective single question. The original survey question reads: “How many times have you borrowed money via online lending platforms and mobile credit applications from 2020 to 2021?”. Respondents choose one answer from eight preset ordered options, which are recoded to range from 0 to 7 for regression estimation: 0 = No use in past 2 years, 1 = 1–2 times, 2 = 3 to 5 times, 3 = 6 to 7 times, 4 = 8 to 10 times, 5 = 11 to 12 times, 6 = 13 to 24 times, 7 = more than 24 times. Larger values correspond to higher frequencies of online credit borrowing. This indicator is treated as an ordinal categorical variable throughout all ordered probit estimations to match its discrete graded measurement structure.

The moderating variable is educational attainment. Consistent with other variable measurement rules, this variable is derived from the original question: “What is your highest level of education?”. The original categorical responses are recoded into years of formal schooling to quantify educational attainment. The specific recoding scheme is as follows: 1 = 0 years (no formal education), 2 = 6 years (primary school), 3 = 9 years (junior high school), 4 = 12 years (senior high school), 5 = 13 years (technical secondary school), 6 = 15 years (junior college), 7 = 16 years (bachelor’s degree), 8 = 19 years (master’s degree), 9 = 22 years (doctoral degree). After transformation, the variable ranges from 0 to 22, where the maximum value of 22 corresponds to respondents with a doctoral degree. Higher values represent higher individual educational attainment, which is linked to stronger information processing capabilities and better financial literacy.

#### 3.2.4 Control variables

Consistent with prior research focusing on individual subjective well-being (Yuan and Bai, 2025; Yang et al., 2026), several individual covariates are added to eliminate confounding factors correlated with core variables and well-being. Referring to mainstream empirical research adopting household micro survey data, some common control variables are embedded into all regression specifications. Variables potentially lying on the pathway are not included to avoid overcontrol. These demographic indicators reflect basic personal traits that may interfere with subjective well-being. The introduction of relevant covariates helps enhance the reliability of model estimation without changing the intrinsic correlation among core research variables. Detailed variable definitions and corresponding descriptive statistics are presented in Table 1.

**Table 1:** Summary statistics of the full analytical sample.

Variables	Definition	Obs	Mean	SD	Min	Max
Subjective well-being	Overall life satisfaction (recoded: 1 = very dissatisfied to 5 = very satisfied).	14,453	3.954	0.827	1	5
Mobile phone dependence	Total daily time spent on 11 mobile phone activities (sum of usage hours).	14,453	6.977	6.558	0	70
Internet credit use	Frequency of using internet credit services (2020–2021, 0 = never to 7 = over 24 times).	14,453	0.341	1.293	0	7
Educational attainment	Years of formal schooling (0 to 22).	14,453	9.960	4.088	0	22
Gender	Gender (1 = male, 0 = female).	14,453	0.688	0.463	0	1
Marital status	Marital status (1 = married, 0 = unmarried).	14,453	0.849	0.358	0	1
Self-rated health	Self-rated health (1 = very poor to 5 = very good).	14,453	3.463	0.986	1	5
Old_ratio	Number of seniors aged over 65 divided by total household population (elderly dependency ratio).	14,453	0.221	0.415	0	1
Risk asset ratio	The proportion of risk assets in total financial assets.	14,453	0.102	0.302	0	1
Risk preference	Risk preference (1 = risk-loving, 0 = risk-averse).	14,453	0.066	0.248	0	1
Phone overuse	Excessive mobile phone use (1 = over 5 h/day, 0 = otherwise).	14,453	0.579	0.494	0	1

Obs, valid sample observations; SD, standard deviation.

Subjective well-being and internet credit use are ordinal graded indicators. Mean and standard deviation are reported only for the unified table layout; distribution proportions rather than central tendency metrics interpret the features of ordinal variables in subsequent regression analyses.

### 3.3 Data analyses

Given that the dependent variable, subjective well-being, is an ordered categorical variable ranging from 1 to 5, ordinary least squares regression is inappropriate for empirical estimation. Therefore, the ordered Probit model is adopted for baseline regression analysis to ensure the accuracy and effectiveness of empirical estimation. The baseline regression model is constructed as follows:

$$\begin{array}{l} \text{Subjective}\_\text{wellbein}g_{i}=\alpha_{0}+\alpha_{1}\text{Mobile}\_\text{phone}\_\text{dependenc}e_{i}+\\ \sum \text{λControl}s_{i}+\varepsilon_{i} \end{array}$$

where Subjective_wellbeing_*i*_ refers to individual subjective well − being, Mobile_phone_dependence_*i*_ represents mobile phone dependence level, *Controls*_*i*_ is a set of individual characteristic control variables, and ε_*i*_ denotes the random error term.

To examine the indirect pathway linking mobile phone dependence to subjective well-being, a mediation analysis is conducted to test whether internet credit use serves as a mediator.

$$\begin{array}{l} \text{internet}\_\text{credit}\_\mathrm{us}e_{i}=\beta_{0}+\beta_{1}\text{Mobile}\_\text{phone}\_\text{dependenc}e_{i}+\\ \sum \text{γControl}s_{i}+\mu_{i} \end{array}$$

$$\begin{array}{l} \text{Subjective}\_\text{wellbein}g_{i}=\delta_{0}+\delta_{1}\text{Mobile}\_\text{phone}\_\text{dependenc}e_{i}+\\ \delta_{2}\text{internet}\_\text{credit}\_\mathrm{us}e_{i}+\\ \sum \text{θControl}s_{i}+\sigma_{i} \end{array}$$

Furthermore, a moderating effect analysis is conducted to examine the moderating role of educational attainment. Interaction terms between mobile phone dependence and educational attainment are added to the baseline model to capture the moderating influence, and the specific moderating effect model is presented as follows:

$$\begin{array}{l} \text{Subjective}\_\text{wellbein}g_{i}=\varphi_{0}+\varphi_{1}\text{Mobile}\_\text{phone}\_{\text{dependence}}_{i}+\\ \varphi_{2}{\mathrm{edu}}_{i}+\varphi_{3}\text{Mobile}\_\text{phone}\_{\text{dependence}}_{i}\\ \times {\mathrm{edu}}_{i}+\sum \text{θControl}s_{i}+\sigma_{i} \end{array}$$

To further examine whether educational attainment moderates the whole mediating path of internet credit use, the bootstrap method proposed by Hayes (2017) is adopted for moderated mediation effect testing. This approach is widely applied in empirical research to judge the significance of conditional indirect effects, which can effectively avoid the statistical defects of traditional stepwise regression and obtain more robust test results for moderated mediation paths.

All estimation routines fully accommodate the ordinal attribute of the internet credit use indicator. Ordered response specifications are applied throughout the whole moderated mediation framework to align with the discrete graded measurement of this core intermediate indicator, eliminating bias brought by mismatched linear model setups for ordinal observed metrics.

## 4 Results

### 4.1 Baseline results

Table 2 presents ordered probit regression outcomes regarding the correlation between mobile phone dependence and subjective well-being, based on 2021 CHFS cross-sectional data. Column (1) conducts a baseline regression without any control variables. Columns (2) further incorporate individual-level and household-level control variables.

**Table 2:** Baseline results.

Variables	(1)	(2)
	Subjective well-being	Subjective well-being
Mobile phone dependence	−0.00555*** (0.00140)	−0.00729*** (0.00148)
Gender		−0.0595*** (0.0200)
Marital status		0.230*** (0.0269)
Self-rated health		0.208*** (0.0102)
Old_ratio		0.359*** (0.0236)
/cut1	−2.571*** (0.0399)	−1.717*** (0.0566)
/cut2	−1.922*** (0.0235)	−1.040*** (0.0471)
/cut3	−0.596*** (0.0150)	0.330*** (0.0445)
/cut4	0.549*** (0.0149)	1.508*** (0.0457)
Observations	14,453	14,453

Standard errors in parentheses.

^*^ *p* < 0.1, ^**^ *p* < 0.05, ^***^ *p* < 0.01.

Regression results show that mobile phone dependence holds a stable negative correlation with subjective well-being at the 1% significance level across both two model settings. After incorporating full sets of confounding controls, the absolute value of the core coefficient rises from −0.00555 in Column (1) to −0.00729 in Column (2). Such numerical variation may reflect the partial mitigation of confounding influences after including observed individual and household characteristics. All control variables follow theoretical expectations. Gender is negatively correlated with subjective well-being, whereas marital status, better self-rated health, and higher old-age dependency ratios are positively linked with subjective well-being, with all coefficients significant at the 1% level. Cut-point parameters also change regularly with the addition of control variables.

Overall, the full sample confirms a steady negative link between mobile phone dependence and subjective well-being. Consistent negative correlation across different regression settings provides empirical evidence for Hypothesis 1, indicating that higher mobile phone dependence corresponds to lower subjective well-being among surveyed consumers.

### 4.2 Robustness checks

Table 3 displays two groups of robustness tests to verify the reliability of baseline regression outcomes. Column (1) adopts a binary dummy variable of mobile phone overuse to replace the original core independent variable. This dummy variable takes the value of one when the original mobile phone dependence index exceeds 5. The corresponding coefficient is still negative and significant at the 1% level, which is consistent with baseline regression conclusions.

**Table 3:** Robustness checks: alternative independent variable and province fixed effects.

Variables	(1)	(2)	(3)	(4)
	Subjective well-being	Subjective well-being	Subjective well-being	Subjective well-being
Mobile phone dependence		−0.00503*** (0.00112)	−0.00840*** (0.00163)	−0.0133*** (0.00305)
Mobile phone dependence squared				0.000239** (0.000107)
Phone Overuse	−0.0596*** (0.0144)			
Controls	Included	Included	Included	Included
Province fixed effects	No	Yes	No	No
*N*	14,453	14,453	14,323	14,453

Column (1) uses a binary indicator of phone overuse. Column (2) includes province-level fixed effects; province dummy coefficients are not reported for brevity. Column (3) trims observations at the 1st and 99th percentiles of the mobile phone dependence index to address extreme value concerns. Column (4) incorporates the squared term of mobile phone dependence to test potential nonlinear associations. Standard errors are reported in parentheses. * *p* < 0.1, ** *p* < 0.05, *** *p* < 0.01.

Column (2) introduces provincial fixed effects to eliminate unobserved regional disparities. The negative correlation between mobile phone dependence and subjective well-being still holds significant at the 1% level. Meanwhile, control variables maintain consistent coefficient signs and significance degrees across different regression models.

Column (3) trims observations below the 1st percentile and above the 99th percentile of the mobile-phone-dependence index to mitigate potential bias driven by extreme values. After removing tail-end observations, the sample size decreases to 14,323. The coefficient of mobile-phone dependence remains significantly negative at the 1% statistical level, with little change in magnitude compared with the baseline estimate. This evidence suggests that the baseline negative association is not dominated by a small number of extreme observations.

Column (4) further examines potential non-linear patterns by adding the squared term of mobile-phone dependence. Both the linear term and squared term are statistically significant. The coefficient signs point to a U-shaped association between mobile-phone dependence and subjective well-being. Nevertheless, the estimated turning point lies in the upper tail of the mobile-phone-dependence distribution. Only a small fraction of respondents falls to the right of this threshold. For most individuals within the sample, higher mobile-phone dependence is still linked to lower subjective well-being.

Collectively, these checks further support the negative correlation proposed in Hypothesis 1 and show that the core research conclusions will not be disturbed by alternative variable measurement methods, regional inherent differences, extreme observations, or potential non-linear relationships.

### 4.3 Mediation and moderation analysis

#### 4.3.1 Mediating role of internet credit use

To identify the transitional linkage running from mobile phone reliance to subjective well-being via internet credit use, the sequential three-column regression procedure proposed by Baron and Kenny (1986) is adopted for mediation assessment. The cross-sectional data used here cannot confirm temporal ordering. What the mediation tests show is whether the observed pattern of associations is consistent with an indirect pathway implied by our hypothesis. They do not identify actual sequential transmission in real life. Table 4 reports mediation regression results to verify the transitional role of internet credit use between mobile phone dependence and subjective well-being, based on 2021 CHFS cross-sectional data. Column (1) replicates the baseline result and verifies the significant negative correlation between mobile phone dependence and subjective well-being at the 1% statistical level.

**Table 4:** Mediation analysis: the role of internet credit use.

Variables	(1)	(2)	(3)
	Subjective well-being	Internet credit use	Subjective well-being
Mobile phone dependence	−0.00729*** (0.00148)	0.0532*** (0.00221)	−0.00556*** (0.00151)
Internet credit use			−0.0407*** (0.00656)
Gender	−0.0595*** (0.0200)	−0.0734** (0.0338)	−0.0610*** (0.0200)
Marital status	0.230*** (0.0269)	−0.149*** (0.0422)	0.227*** (0.0269)
Self-rated health	0.208*** (0.0102)	0.0599*** (0.0164)	0.209*** (0.0102)
Old_ratio	0.359*** (0.0236)	−0.925*** (0.0691)	0.350*** (0.0236)
/cut1	−1.717*** (0.0566)	1.742*** (0.0703)	−1.722*** (0.0566)
/cut2	−1.040*** (0.0471)	1.957*** (0.0707)	−1.045*** (0.0471)
/cut3	0.330*** (0.0445)	2.093*** (0.0710)	0.327*** (0.0445)
/cut4	1.508*** (0.0457)	2.153*** (0.0711)	1.507*** (0.0457)
/cut5		2.232*** (0.0718)	
/cut6		2.415*** (0.0730)	
/cut7		2.517*** (0.0735)	
Observations	14,453	14,453	14,453

Robust standard errors in parentheses.

*** *p* < 0.01, ** *p* < 0.05, * *p* < 0.1.

Column (2) captures the correlation between mobile phone dependence and the mediating variable. Mobile phone dependence presents a significant positive correlation with internet credit use at the 1% level. Column (3) incorporates both mobile phone dependence and internet credit use into one regression model. Mobile phone dependence still holds a negative correlation with subjective well-being, and internet credit use is also negatively linked with subjective well-being with statistical significance.

After adding internet credit use, the absolute value of the mobile phone dependence coefficient declines visibly. Combined with significant coefficients in Column (2) and Column (3), the statistical outcomes are consistent with the presence of an indirect empirical pattern. The changing coefficient trend across the three regression columns meets the standard judgment criteria for mediating paths. The empirical results demonstrate that internet credit use statistically accounts for part of the observed association. Higher mobile phone dependence is associated with more frequent internet credit use, which in turn correlates with lower subjective well-being. However, the sample-average mediating estimate cannot be generalized uniformly to every individual in the full dataset. These empirical results provide empirical support for Hypothesis 2.

Within the analytical sample, 90.79% of respondents reported no internet credit use over the past two-year period. To test whether mediation findings are influenced by the heavily skewed distribution of internet credit use, internet-credit-use is recoded into a binary indicator labelled “ever_icu.” In this measure, a value of 1 represents respondents with any history of online borrowing, while 0 refers to individuals who have never accessed internet-credit services. The mediation model is re-estimated. Outcomes stay qualitatively unchanged. Mobile phone dependence shows a positive correlation with this binary ever-used measure. Meanwhile, the binary internet-credit indicator correlates negatively with subjective well-being, and the indirect association retains statistical significance. This sensitivity check demonstrates that the core mediating pattern does not solely stem from the original ordered frequency measure. Complete estimation results are available in Table A1.

#### 4.3.2 The moderating role of educational attainment in the mediation of internet credit use

Table 5 reports the outcomes of moderated mediation analysis, which targets the conditioning role of educational attainment in the internet credit-based mediating pathway. This empirical test adopts the bootstrap method put forward by Hayes (2017), with 1,000 repeated sampling times to construct bias-corrected confidence intervals.

**Table 5:** Bootstrap test of moderated mediation in the association of mobile phone dependence with subjective well-being.

Stratified level of educational attainment	Indirect interconnection coefficient	Bootstrapped standard error	Bootstrapped 95% confidence interval	
Low (mean minus 1 SD)	−0.00485*	0.00109	−0.0075	−0.00308
Mean	−0.00641*	0.00121	−0.00906	−0.0044
High (mean plus 1 SD)	−0.00798*	0.0015	−0.01129	−0.00542
Conditioning index of moderated mediation	−0.00156*	0.00049	−0.00284	−0.00081

This table reports bootstrapped results for the conditional indirect interconnection running from mobile phone reliance to subjective well-being via internet credit use, with educational attainment set as the conditioning variable. The three stratified levels correspond to values at one standard deviation below the sample mean, the sample mean, and one standard deviation above the sample mean of educational attainment. The conditioning index quantifies the degree of layered differentiation across indirect interconnections. Bootstrapped standard errors are displayed in the third column, and bias-corrected 95% confidence intervals are listed in the final column. An asterisk * marks statistical significance at the 5% threshold; intervals that exclude zero confirm meaningful statistical correlation.

The indirect empirical pattern from mobile phone dependence to subjective well-being through internet credit use remains statistically detectable at the 1% level across groups with different educational backgrounds. For respondents with low educational attainment, the indirect coefficient is −0.00485, and the corresponding 95% bias-corrected confidence interval ranges from −0.00750 to −0.00308. For samples with average educational attainment, the indirect coefficient turns out to be −0.00641, with a 95% confidence interval covering [−0.00906, −0.00440]. For consumers with high educational attainment, the indirect coefficient reaches −0.00798, and the 95% confidence interval is [−0.01129, −0.00542].

The absolute value of the indirect coefficient rises steadily along with the improvement of educational level, suggesting that the mediating pathway becomes stronger among better-educated groups. The moderation index equals −0.00156, with a 95% bias-corrected confidence interval of [−0.00284, −0.00081]. Since the confidence interval excludes zero, statistical results support a conditioning role of educational attainment in this mediating pathway.

Combined with empirical evidence for Hypotheses 1 and 2 obtained in prior regression tests, the changing trend of indirect linkage fits the expectation of Hypothesis 3 perfectly. The indirect connection between mobile phone dependence and subjective well-being via internet credit use is strengthened among consumers with higher educational attainment. All estimation results offer reliable empirical evidence for the whole moderated mediation model constructed in this research.

#### 4.3.3 Heterogeneity analysis by risk asset holdings

Table 6 shows heterogeneity estimation results grouped by household risk asset holdings, exploring whether the core correlation differs among households with diverse financial asset structures. Based on household risk asset holding levels, the full 2021 CHFS cross-sectional sample is split into two subgroups, namely the low-risk asset holdings group in Column (1) and the high-risk asset holdings group in Column (2).

**Table 6:** Risk asset holdings heterogeneity.

Variables	(1)	(2)
	Low-risk-asset group: subjective well-being	High-risk-asset group: subjective well-being
Mobile phone dependence	−0.00515*** (0.00115)	−0.00574 (0.00402)
Gender	−0.0435*** (0.0156)	−0.0579 (0.0460)
Marital status	0.166*** (0.0214)	0.242*** (0.0648)
Self-rated health	0.154*** (0.00788)	0.151*** (0.0237)
Old_ratio	0.259*** (0.0174)	0.241*** (0.0727)
_cons	3.294*** (0.0356)	3.203*** (0.0972)
*N*	12,981	1,472

Standard errors in parentheses.

* *p* < 0.1, ** *p* < 0.05, *** *p* < 0.01.

Column (1) displays regression outcomes for households with low-risk asset holdings. The coefficient of mobile phone dependence is significantly negative at the 1% level, which is consistent with the baseline correlation summarized in Hypothesis 1. For households with limited participation in risky financial markets, mobile phone dependence exhibits a negative association with subjective well-being.

Column (2) presents results for households with high-risk asset holdings. Though the coefficient of mobile phone dependence still keeps a negative sign, it fails to pass conventional significance tests. It means the negative linkage between mobile phone dependence and subjective well-being is not prominent for households with abundant risk asset allocation.

Households with higher risk asset holdings tend to have greater financial literacy and more diversified coping strategies. These resources may offset some of the psychological costs associated with mobile dependence. Households with fewer risk assets lack such buffers, which may explain why their well-being shows a stronger negative association with mobile phone use. The coefficient for the low-risk-asset group is −0.00515 (*p* < 0.01), compared with −0.00574 (*p* > 0.10) for the high-risk-asset group. This pattern is consistent with a resource-buffer interpretation, though the substantial imbalance in group sizes (12,981 vs. 1,472) cautions against overinterpretation of the non-significant estimate in the smaller group.

These heterogeneous results supplement differentiated empirical evidence for Hypothesis 1. The negative correlation between mobile phone dependence and subjective well-being is more prominent among households with low-risk asset holdings, while it is not statistically detectable in the smaller high-risk-asset group.

#### 4.3.4 Heterogeneity analysis by risk preference

Table 7 presents heterogeneity regression results grouped by individual risk preference, to compare differences in the core correlation and its mediating pathway among respondents with different risk attitudes. Based on 2021 CHFS cross-sectional data, the full sample is divided into two subgroups: the low-risk preference group in Column (1) and the high-risk preference group in Column (2).

**Table 7:** Risk preference heterogeneity.

Variables	(1)	(2)
	Low-risk preference group: subjective well-being	High-risk preference group: subjective well-being
Mobile phone dependence	−0.00364*** (0.00119)	−0.00239 (0.00386)
Internet credit use	−0.0310*** (0.00544)	−0.0211 (0.0135)
Gender	−0.0396*** (0.0152)	−0.113* (0.0612)
Marital status	0.160*** (0.0210)	0.323*** (0.0815)
Self-rated health	0.155*** (0.00770)	0.151*** (0.0314)
Old_ratio	0.258*** (0.0172)	0.178** (0.0829)
_cons	3.291*** (0.0343)	3.153*** (0.147)
*N*	13,503	950

Standard errors in parentheses.

* *p* < 0.1, ** *p* < 0.05, *** *p* < 0.01.

Column (1) reports results of risk-averse individuals. Mobile phone dependence has a significantly negative correlation with subjective well-being at the 1% level, which is consistent with the baseline correlation stated in Hypothesis 1. Internet credit use also shows a significant negative correlation with subjective well-being at the same significance level. The results verify the mediating pathway proposed in Hypothesis 2. For risk-averse individuals, higher mobile phone dependence correlates with lower subjective well-being, and internet credit use is consistent with a partial intermediate associative pattern between the two variables.

Column (2) shows regression outcomes of individuals with high-risk preference. Both mobile phone dependence and internet credit use carry negative coefficients, yet neither variable achieves statistical significance. It indicates that the negative correlation between mobile phone dependence and subjective well-being, together with the mediating pathway of internet credit use, cannot be observed among consumers with high risk tolerance.

Subgroup results reveal evident disparities. Compared with those holding risky assets, risk-averse respondents are more susceptible to financial pressure and life instability stemming from heavy smartphone reliance and internet credit use. This coincides with a more pronounced negative association between these behaviors and subjective well-being. By contrast, people with higher risk tolerance adapt better to digital financial activities, which corresponds to a weaker above correlation and its indirect transmission pathway. According to Behavioral Decision Theory, risk-averse people react more strongly to financial uncertainty and debt pressure. They are likely to develop more negative views of potential financial risks brought by over-reliance on mobile phones and online credit. Risk-tolerant individuals, however, respond less to these financial uncertainties, which corresponds to a weaker observed association. However, the high-risk subgroup has far fewer observations than the low-risk group. Such sample imbalance lowers statistical power. Hence, insignificant coefficients within the high-risk subgroup cannot be interpreted as proof that no association exists. Future research with larger, more balanced samples is required to validate these findings.

This set of heterogeneity results clarifies the boundary conditions of Hypothesis 1 and Hypothesis 2. Both the negative correlation between mobile phone dependence and subjective well-being and the mediating pathway of internet credit use are only significant among individuals with low-risk preference.

## 5 Discussion

The present work explores the interconnection between mobile phone reliance and consumers’ subjective well-being, alongside the transitional route associated with internet credit participation and the conditioning role played by educational attainment. All analytical procedures draw on single-year cross-sectional records from the 2021 China Household Finance Survey. Steady and matching results can be observed throughout baseline estimation, transitional channel tests, conditional intermediate analysis, group differentiation tests, and stability verification procedures.

The baseline results confirm the negative association between mobile phone dependence and subjective well-being, supporting Hypothesis 1. The negative relevance persists across diverse model specifications and after absorbing individual and household characteristic variables, revealing that deeper reliance on mobile devices coincides with poorer subjective well-being among consumers. This empirical rule echoes the viewpoint proposed by Nie et al. (2021), who record that stronger smartphone usage intensity correlates with lower subjective well-being, especially for residents spending over 3 h on mobile terminals each day. Consistent with this finding, existing research targeting college students also verifies the negative correlation between smartphone addiction and individual subjective well-being (Su and He, 2024). Such adverse psychological changes mainly stem from long-term virtual immersion and fragmented information stimulation, which destroy the balance between offline social interaction and digital leisure (Donoso et al., 2021).

It is noteworthy that the correlation between digital behavior and subjective well-being holds obvious duality, which explains the contradictory conclusions in existing empirical studies. Some scholars document a positive relationship between smartphone usage and well-being among teenagers, arguing that longer duration and higher frequency of smartphone use correspond to higher subjective well-being among teenagers (Marciano et al., 2022). Similarly, mobile internet use can lift subjective well-being among Chinese elderly residents (Lu and Kandilov, 2021), and general internet use can improve residents’ overall welfare by strengthening individual self-efficacy, optimizing access to health information, and expanding non-agricultural employment opportunities (Shuai et al., 2025). Digital embedding also brings welfare growth among rural farmers (Chen et al., 2026). Nevertheless, traditional wired internet presents more positive relevance to subjective well-being compared with mobile internet (Tan et al., 2024). Such divergent findings can be attributed to heterogeneous usage patterns: internet use only connects with lower subjective well-being under problematic usage conditions, while rational internet use links with improved well-being (Donoso et al., 2021). Besides individual usage differences, digital infrastructure expansion also brings a sustained decline in self-rated happiness among rural residents (Fang et al., 2026).

In addition, digital-financial behaviors associated with excessive mobile phone use complicate the above correlation. As a typical cashless payment tool embedded in smartphones, mobile payment is associated with excessive consumption behaviors and lower subjective well-being (Wang et al., 2022), while a reasonable mobile payment application also correlates with improved subjective well-being (Zhao et al., 2024). Digital financial services show dual characteristics in adjusting individual financial perceptions: they weaken the correlation between household financial behaviors and subjective financial status and, meanwhile, relieve the negative relevance of financial pressure on personal perceived financial conditions (Mahdzan et al., 2023).

Mediation analysis further shows an empirical pattern consistent with internet credit use statistically accounting for part of the association between mobile phone dependence and well-being. The statistical outcomes indicate that mobile phone dependence correlates with more frequent internet credit participation, and such financial behavior in turn is associated with lower subjective well-being. This observed correlational pattern provides empirical support for Hypothesis 2 and clarifies a hidden behavioral channel through which digital activities are associated with individual welfare within the household finance framework. The formation logic of this channel can be explained by the operational characteristics of big-data credit platforms summarised by Zhang et al. (2015). Big data empowers online credit services from multiple dimensions: massive user data expands lenders’ access to potential borrowers, accurate risk prediction cuts operational costs of lending institutions, diversified services meet differentiated demands of financial platforms, and complete information protection measures maintain sustainable operation of online credit businesses. Greater exposure to credit-marketing information via mobile terminals correlates with reduced barriers to online borrowing and coincides with impulsive loan-related demands.

From the perspective of financial risk spillover, internet access raises household credit default risks; households with internet access record 0.474 times higher default probability than households without internet access at the sample mean level (Donou-Adonsou and Basnet, 2019). Accelerated digital transformation increases consumers’ access to high-cost payday loans and raises risks of spiralling household debt (Chen, 2020). The rapid development of internet credit services has caused prominent debt risks and a series of negative psychological and social problems, especially among young groups (Ma and Yao, 2023). Such sequential transmission logic shares consistent analytical thinking with the research recorded by Nie et al. (2021). Their study verifies that agricultural and non-agricultural income jointly form an intermediate bridge linking smartphone usage to subjective well-being. The parallel analytical thinking implies that various economic and financial channels can act as transitional routes through which digital activities are associated with changes in consumers’ subjective welfare status.

The moderated mediation results are consistent with educational attainment being associated with the strength of this indirect empirical pattern. The indirect linkage running from mobile phone dependence to subjective well-being via internet credit use is stronger among consumers with higher educational attainment. The obvious gradient difference of coefficients across educational subgroups and the significant moderating statistic offer reliable support for Hypothesis 3.

Firstly, educational background has a direct correlation with individual subjective well-being. Existing studies confirm a positive correlation between educational attainment and subjective well-being (Jin et al., 2020), and educational level presents positive relevance to residents’ subjective well-being (Dong et al., 2025). Residents with higher education show higher average subjective well-being than those without higher education, and educational level can positively predict individual subjective well-being (Qin et al., 2025). Meanwhile, subjective well-being varies significantly across educational groups, with undergraduates having higher well-being levels than junior college students and postgraduates (Su and He, 2024). Secondly, education can optimize individual cognition and comprehensive capabilities and further promote the development of green digital finance (Lee et al., 2025).

Thirdly, education brings systematic differentiation to consumers’ digital financial responses. Digital finance affects residents’ subjective well-being differently across groups divided by educational attainment, urban–rural attributes, and consumption patterns (Wang and Li, 2026). Digital finance is associated with lower individual happiness through increased household debt burdens and excessive consumption (Meng and Xiao, 2023). Meanwhile, the welfare relevance of digital embedding presents evident heterogeneity, with more significant correlations among western rural residents, low-income groups, residents with a basic education background, and young and middle-aged groups (Chen et al., 2026).

Such differentiated responses are rooted in individual digital literacy and financial capacity gaps brought about by education. Residents with sound digital literacy can operate mobile payment and mobile banking systems more smoothly and perceive higher convenience brought by network technologies (Ullah et al., 2022). Relevant research further supplements that individual financial knowledge is essential for household financial decisions, while financial knowledge alone cannot guarantee matching financial skills. Individuals can accumulate financial skills through market observation, practical transaction experience, and peer learning, including budget planning via mobile applications and basic banking service cognition (Ullah et al., 2022). Residents equipped with adequate financial skills can better recognize the practical value of digital financial products and perceive more benefits brought by mobile financial services (Ullah et al., 2022). In addition, financial service knowledge acts as a core determinant of residents’ access to fintech services, and residents’ degree of cognition of relevant financial rules greatly affects their participation in digital financial services (Hasan et al., 2023). Therefore, well-educated consumers with stronger digital literacy and financial capabilities are more likely to receive mobile credit advertisements and engage in impulsive online borrowing behaviors. In contrast, consumers with lower educational attainment suffer from an insufficient digital foundation, face obvious obstacles in using digital credit products, and show weaker responses to mobile financial information stimulation.

Stratified examinations based on risk asset holdings and personal risk preferences offer supplementary evidence regarding the boundary scope of the core linkages uncovered in this work. The negative tie connecting mobile phone dependence to subjective well-being, together with the transmission path built through internet credit participation, is statistically detectable only among consumers with limited risk asset holdings and low tolerance towards financial risks. For populations equipped with ample risk asset allocation or higher willingness to bear financial risks, the above two sets of ties turn weaker and fail to pass significance tests.

Such group disparities conform to the core reasoning of Behavioral Decision Theory. Groups inclined to avoid risks tend to feel heavier pressure from debt and greater unease facing financial uncertainties. Unregulated online borrowing behaviors are associated with more severe psychological disturbance among these groups. Digital financial services can ease part of the financial burden felt by risk-averse residents (Mahdzan et al., 2023), yet such relief cannot counteract the lasting adverse psychological fluctuations brought by excessive online borrowing derived from heavy mobile phone reliance.

A series of stability tests are carried out, which cover revised measurement standards for core variables and additional confounding factors added to regression equations. All core discovered linkages remain unchanged across these adjusted empirical frameworks. Consistent correlation tendencies observed under diverse regression setups strengthen the reliability of results from baseline estimation, intermediate channel tests, and moderated transmission path analysis.

Overall, the derived outcomes expand existing understanding of how mobile phone reliance interacts with consumers’ subjective well-being within the digital finance context. Observing internet credit use as a plausible correlational transmission pattern and educational attainment as a conditioning factor lays out a multi-level interactive logic running through digital usage habits, household financial engagement, and individual subjective welfare. Stratified test results further suggest that the tightness of these interconnections is bounded by household asset portfolios and individual risk attitudes. The recorded patterns provide detailed empirical reference for follow-up theoretical extension and differentiated policy arrangements targeting digital financial governance.

## 6 Conclusion and future research agenda

### 6.1 Conclusion

Using cross-sectional data from the 2021 China Household Finance Survey, this study systematically explores the association between mobile phone dependence and consumers’ subjective well-being, together with the mediating role of internet credit use and the moderating role of educational attainment. Multiple empirical strategies, including baseline regression, mediation analysis, moderated mediation test, heterogeneity analysis, and robustness checks, are applied to verify the proposed hypotheses.

The baseline outcomes reveal a notable negative association between mobile phone dependence and subjective well-being, which remains stable after controlling for individual and household characteristics. This empirical pattern fully supports Hypothesis 1.

Mediation analysis yields results consistent with internet credit use statistically accounting for part of the observed association. Higher mobile phone dependence corresponds to more frequent use of internet credit services, and such financial behavior further links to lower subjective well-being. This observed empirical pattern provides empirical support for Hypothesis 2.

The moderated mediation results indicate that educational attainment shows a significant moderating association in this indirect empirical pattern. The indirect association running from mobile phone dependence to subjective well-being via internet credit use becomes stronger among consumers with higher educational attainment, which is consistent with the expectation of Hypothesis 3.

Heterogeneity examinations based on risk asset holdings and risk preferences suggest distinct group differences. The core correlation and mediating pathway are statistically significant among groups with low-risk asset holdings and low-risk preferences, while these relationships become insignificant for groups holding more risk assets or bearing higher risk tolerance. Robustness tests with alternative variable settings further support that all core findings are reliable and not sensitive to model adjustments.

In general, this study provides evidence on the mediating role of internet credit use and group differences in the association between mobile phone dependence and subjective well-being within the digital finance context. It supplements empirical evidence for the interactive relationship among digital behavior, financial participation, and individual well-being.

### 6.2 Policy implications

The empirical findings deliver practical implications for governing digital behavior, regulating internet credit services, and improving consumers’ well-being.

First, targeted guidance on rational mobile phone use is necessary. Relevant departments and communities could launch public education campaigns to help consumers recognize the potential drawbacks of excessive mobile phone reliance and advocate healthy digital lifestyles. Such guidance is especially essential for groups with low risk tolerance, who are more susceptible to the negative correlates of mobile phone dependence.

Second, stricter supervision over internet credit businesses should be strengthened. Platforms need to standardize marketing strategies and credit approval rules to mitigate impulsive applications and excessive borrowing associated with mobile scenarios. On the marketing side, inducements such as “one-click borrowing” and “instant approval without review” should be restricted. On the credit evaluation side, a rational credit limit management system should be established to avoid over-lending to specific groups, thereby alleviating the psychological burden associated with excessive debt.

Third, differentiated financial protection and education strategies are required for consumers with varied educational backgrounds. It is worth noting that many internet credit platforms tend to grant higher credit limits and more lenient borrowing conditions to highly educated users—who are often perceived as “prime borrowers.” However, our empirical findings reveal that the indirect negative association between mobile phone dependence and subjective well-being via internet credit use is actually stronger among highly educated groups. In other words, greater credit accessibility is associated with heightened vulnerability among this group to adverse outcomes of excessive borrowing that coincides with higher mobile-phone dependence. Therefore, rather than relying solely on simple “risk warnings” or moral suasion, regulators should promote the adoption of behavioral intervention tools in credit evaluation and marketing practices targeting highly educated consumers. Specifically, (1) Regulators could encourage the development of customized compliance-based credit products for highly educated groups, with clearly specified credit limits, annualized percentage rates (APR), and repayment terms. This approach would eliminate hidden fees and set reasonable credit thresholds aligned with educational background and income levels to avoid over-indebtedness. (2) Platforms should be required to adopt transparent information disclosure mechanisms for all credit products targeting highly educated groups. Such mechanisms would prominently display the true comprehensive annualized cost, delinquency consequences, and early repayment rules at the point of application, while prohibiting misleading marketing claims such as “low interest” or “zero threshold.” (3) Regulators could implement dynamic credit monitoring and early warning systems for highly educated users, detecting multi-platform borrowing and frequent credit applications. These systems would guide users to keep their outstanding loan accounts within a reasonable range, thereby preventing negative impacts on their credit records. (4) Platforms could introduce personalized credit cap alerts that trigger non-binding reminders when a user’s cumulative borrowing approaches a reasonable threshold corresponding to their income level. This approach provides decision-relevant information rather than prescriptive restrictions, preserving consumer autonomy while encouraging prudent borrowing.

For groups with lower educational attainment, regulators should prioritize the dissemination of basic financial knowledge and the cultivation of risk identification skills. Simplified visual materials, short-video courses, and community-based financial literacy programs could help establish rational consumption and borrowing attitudes, reducing blind participation in digital credit services.

Fourth, financial service design should account for individual differences in risk attitudes. For risk-averse consumers, more inclusive and prudent financial products could be offered—such as default lower credit limits or opt-out savings mechanisms—to avoid exacerbating their psychological burden while ensuring financial accessibility.

### 6.3 Limitations and future research agenda

This study has several limitations that leave room for further exploration. First, this study relies on cross-sectional data from a single survey wave, which limits the ability to establish causal ordering. The empirical tests are designed to check whether the observed patterns are consistent with theoretically derived associative hypotheses, rather than to identify causal effects. Despite the inclusion of multiple control variables and the performance of various robustness checks, the possibility of omitted variable bias or reverse causality cannot be completely ruled out. Unobserved attributes such as self-control, time preference, and personality traits may confound the reported relationships. Reverse causality also cannot be ruled out. For example, lower subjective well-being could be associated with heavier mobile phone use, rather than the reverse. The results should therefore be interpreted as correlational evidence that is consistent with the proposed theoretical framework, not as definitive causal estimates. Dynamic changes in the focal variables over time are not captured in the current design, which further constrains causal inference. Second, the measurement of core variables follows conventional standards, while potential latent characteristics and detailed behavioral patterns are not fully explored. Third, this study takes educational attainment, risk asset holdings, and risk preference as grouping indicators for heterogeneity analysis, and other personal and family attributes are not discussed. Fourthly, the heterogeneity analyses are constrained by sample size imbalance across subgroups, particularly for risk preference (13,503 vs. 950). This imbalance limits statistical power to detect potentially meaningful differences in the smaller groups. On the basis of current research, several directions can be pursued in follow-up studies.

Firstly, longitudinal panel data can be adopted to track the dynamic evolution of key variables. Combined with advanced identification strategies, subsequent research can better distinguish causal links rather than simple correlation.

Secondly, multi-dimensional measurement of core variables can be constructed. Fine-grained classification of mobile phone usage scenarios and internet credit behaviors helps reveal more detailed transmission rules.

Thirdly, future research could extend the heterogeneity analysis by examining whether the observed relationships differ across other sociodemographic dimensions, such as age groups, urban and rural areas, occupational types, and family structures, so as to further enrich the understanding of boundary conditions. Studies with larger and more balanced samples are also encouraged to validate the subgroup patterns identified in the present study.

Fourthly, more intermediary and moderating factors can be incorporated into the analytical framework. Factors such as social interaction, financial literacy, and consumption habits can be included to build a more complete theoretical framework for relevant research fields.

## Data Availability

The original contributions presented in the study are included in the article/supplementary material, further inquiries can be directed to the corresponding author.

## Appendix

**Table A1:** Mediation analysis: the role of binary-coded internet credit use.

Variables	(1)	(2)	(3)
	m0	m1	m2
	happiness	ever_icu	happiness
mobilephonedependence	−0.00729*** (0.00148)	0.0995*** (0.00447)	−0.00472*** (0.00152)
ever_icu			−0.226*** (0.0307)
gender	−0.0595*** (0.0200)	−0.122* (0.0679)	−0.0613*** (0.0200)
marriage	0.230*** (0.0269)	−0.340*** (0.0844)	0.224*** (0.0269)
health	0.208*** (0.0102)	0.106*** (0.0327)	0.210*** (0.0102)
old_ratio	0.359*** (0.0236)	−2.089*** (0.174)	0.345*** (0.0237)
/cut1	−1.717*** (0.0566)		−1.729*** (0.0567)
/cut2	−1.040*** (0.0471)		−1.051*** (0.0471)
/cut3	0.330*** (0.0445)		0.322*** (0.0444)
/cut4	1.508*** (0.0457)		1.502*** (0.0456)
Constant		−3.007*** (0.139)	
Observations	14,453	14,453	14,453

Column (1) presents the ordered probit model for subjective well-being. Column (2) Logit model for binary mediator ever_icu; Column (3) presents the ordered probit model for subjective well-being, which includes both mobile phone dependence and the mediator ever_icu. *** *p* < 0.01, ** *p <* 0.05, * *p* < 0.1.
